# Frequency of minimal hepatic encepalopathy in illeterate patients with compensated cirrhosis

**DOI:** 10.12669/pjms.323.9655

**Published:** 2016

**Authors:** Bader Faiyaz Zuberi, Haris Alvi, Faisal Faiyaz Zuberi, Tazeen Rasheed, Zunaira Nawaz, Fatima-tuz-Zohra

**Affiliations:** 1 Prof. Bader Faiyaz Zuberi, FCPS. Department of Medicine, Dow University of Health Sciences, Karachi, Pakistan; 2 Dr. Haris Alvi, FCPS. Department of Medicine, Dow University of Health Sciences, Karachi, Pakistan; 3 Dr. Faisal Faiyaz Zuberi, FCPS. Department of Pulmonology, Dow University of Health Sciences, Karachi, Pakistan; 4 Dr. Tazeen Rasheed, FCPS. Department of Medicine, Dow University of Health Sciences, Karachi, Pakistan; 5 Dr. Zunaira Nawaz, FCPS. Department of Medicine, Dow University of Health Sciences, Karachi, Pakistan; 6 Dr. Fatima-tuz-Zohra, MBBS. Department of Medicine, Dow University of Health Sciences, Karachi, Pakistan

**Keywords:** Minimal Hepatic Encephalopathy, Illetrate, Compensated Cirrhosis

## Abstract

**Objective:**

To determine frequency of Minimal Hepatic Encephalopathy in illiterate patients with compensated cirrhosis.

**Methods:**

Illiterate patients with compensated cirrhosis with F4 Score on Shear-wave Elastography were selected for study after informed consent. Sample size was estimated at 106. Selected patients were subjected to two tests for detection of MHE, Number Connection Test A and Block Design Test. Patients taking ≥ 30 seconds were labelled as Positive for MHE.

**Result:**

Out of 110 selected patients 10.9% were alcoholics and in 8.2% of patients no hepatic virus infection was detected. HCV was positive in 48.2% patients while HBV was positive in 13.6% of patients. MHE was detected in 72 (65.5%) of patients. Major differences were found in MHE Stage II & III by two tests. Over all BDT detected more cases and gave higher Staging in Stage II & III as compared to NCT-A test.

**Conclusion:**

Minimal Hepatic Encephalopathy (MHE) could be detected in illiterate patients using NCT-A and BDT Tests.

## INTRODUCTION

Hepatic Encephalopathy (HE) is a common and frequent complication of cirrhosis.^1^ It continues to remain as a major complication in patients with chronic liver disease despite advances in management of liver diseases during last decade. About 30-45% of patients with cirrhosis develop HE which ranges from mild symptoms like easy fatigue and sleep inversion to full blown coma.^2^ HE is associated with reduced survival even after its single episode.^3^ Minimal Hepatic Encephalopathy (MHE) is a complication occurring in cirrhosis due to neuro-cognitive dysfunction. It is characterized by specific complex cognitive dysfunction which is independent of sleep dysfunction or problems with overall intelligence. These cognitive shifts are missed during routine clinical examination and identified uniquely through psychometric or neurological tests.^4^,^5^ Although named “minimal”, it has far reaching impact on quality of life, ability to function in daily life and can progress to overt hepatic encephalopathy.^4^ The prevalence of MHE in HE is reported as 30-84%.^5^ It not only causes deterioration in quality of life but impairs their ability to drive and operate machinery due to attention deficits.^5^,^6^ Never the less MHE is a treatable and reversible stage of HE,^7^ and thus it is prudent, to identify MHE to optimize treatment and prognosis.^8^

Although patients with HE have impaired intellectual functioning, personality changes, altered level of consciousness, and neuromuscular dysfunction; patients with MHE have no recognizable clinical symptoms, but do have mild cognitive and psychomotor deficits. There is no “gold standard” test for determining MHE. Neuropsychological (NP) and neurophysiological methods have been used tests to diagnose this condition.^9^,^10^ Due to its obscure nature, its impact on Quality of Life (QoL) remained undetermined for long. Later on recently many studies have documented its negative effects on QoL of patients, their ability to do their jobs and intellectual functions and their driving capabilities.^2^,^11^ Many abnormalities in brain white matter have been identified including low-grade edema and structural impairments visible by MR imaging and are thought to be responsible for poor neurological performance and dysfunctions.^8^

Although some work on MHE has been reported from India, none to the best of our knowledge, has reported it in illiterate patients from our region, and the extent of this disorder remains to be documented for proper management to increase not only quality of life in such patients but also to prevent their progression to overt HE. Objective of this study was to determine frequency of Minimal Hepatic Encephalopathy in illiterate patients with compensated cirrhosis.

## METHODS

Study was conducted in Hepatitis Clinic at Civil Hospital Karachi between the period of May 2014 and April 2015. Patients reporting with relapse, non-response to standard or Pegylated interferon and naïve patients of hepatitis were selected for initial evaluation. Illiterate patients with compensated cirrhosis (no ascites, overt encephalopathy, variceal bleeding or jaundice) with F4 Score on Shear-wave Elastography were selected for study after informed consent. Patients without primary education (less than five years of school) were taken as illiterate. Patients with complete Quranic education were taken as literate. Patients on diuretics and with history of overt HE or variceal bleed or band ligation, active alcohol intake, psychotropic drug usage, and those with structural brain damage were excluded.

Sample size estimation was done by PASS 13 Software using Wilson Score Method for upper one-sided interval type. Confidence interval (1-alpha) was kept at 0.95, one-sided precision was kept at 0.05. Using proportion of 84%^5^ the sample size was estimated at 106.

Demographic data of selected patients was recorded; note was made of alcohol consumption. Selected patients were subjected to two tests for detection of MHE, Number Connection Test A (NCT-A) and Block Design Test (BDT). Each patient undertook test for three times and result of best of three was taken. Patients taking less than 30 seconds in each test to complete were labelled as Negative for MHE. Patients taking ≥ 30 seconds were labelled as Positive for MHE. Their further staging was done as under:

**Table:** 

< 30 seconds	Absent
31-50 seconds	Stage-I
51-80 seconds	Stage-II
81-120 seconds	Stage-III
> 120 seconds	Stage-IV
	

Mean age±SD was reported according to gender and compared using Student’s t-test. Frequencies of underlying causes of cirrhosis were reported. Frequency of MHE was reported in percentages and compared with stages of MHE using χ^2^ test.

## RESULTS

Applying the selection criteria 110 patients were selected for study. These included 63 (57.3%) males and 47 (42.7%) females. Mean age ±SD of males was 39.9 ±9.5 years while that of females was 36.0 ±10.0 years. Mean age of females was statistically lower than that of males with p = 0.046. Out of 110 selected patients 10.9% were alcoholics and in 8.2% of patients no hepatic virus infection was detected. HCV was positive in 48.2% patients while HBV was positive in 13.6% of patients. The demographic details of selected patients are given in Table-I.


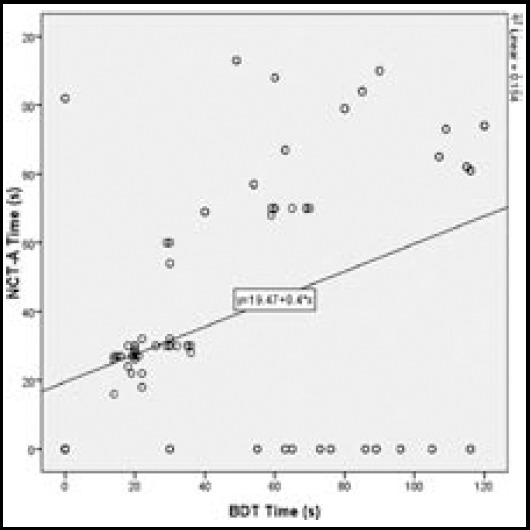


**Table-I:** Demographic Details of Selected Patients.

*Gender*						*Total n (%)*

		*Male*		*Female*			

		*Count*	*Column N %*	*Count*	*Column N %*		
Virus	No Virus	6	9.2%	3	6.7%	9 (8.2%)
	HCV	33	50.8%	20	44.4%	53 (48.2%)
	HBV	7	10.8%	8	17.8%	15 (13.6%)
	HBV + HDV	7	10.8%	5	11.1%	12 (10.9%)
	HCV + HBV	7	10.8%	5	11.1%	12 (10.9%)
	HCV + HBV + HDV	5	7.7%	4	8.9%	9 (8.2%)
Alcoholic	10	15.4%	2	4.4%	12 (10.9%)
Mean Age ±SD years		39.9 ±9.5		36.0 ±10.2		38.3 ±10.0

Mean time taken for NCT-A test was 38.6 ±38.1 sec while mean time for BDT was 47.6 ±37.2 sec. Comparing times of both test by ‘Paired Sample t-Test’ showed that BDT time was significantly more than NCT-A test with p =0.025 and 95% CI of -16.8 to -1.12. Time of both tests showed poor linear correlation with each other (r^2^=0.154; p < 0.001) showing that statistically significant difference in time correlations occurred in the two methods used in this study (Fig.1).

Patients were segregated into two groups on the basis of test results of both test using cut off of 30 seconds to determine the frequency of MHE. According to NCT-A test 79 (71.8%) of patients were having MHE while according to BDT, 77 (70.0%) of patients were having MHE. Both tests were positive in 72 (65.5%) of patients and were labelled as having MHE according to study protocol. Details are given in Table-II.

**Table-II:** MHE frequencies by NCT-A & BDT Tests.

			*BDT Status*		*Total*

			*Negative*	*Positive*	
NCT-A Status	Negative	Count	26	5	31
		% of Total	23.6%	4.5%	28.2%
	Positive	Count	7	72	79
		% of Total	6.4%	65.5%	71.8%
Total		Count	33	77	110
		% of Total	30.0%	70.0%	100.0%

Staging of MHE on basis of two tests time scores is given in Table-III. Major differences were found in MHE Stage II & III by two tests. Over all BDT detected more cases and gave higher Staging in Stage II & III as compared to NCT-A test.

**Table-III:** Patient Staging of MHE according to NCT-A & BDT Tests.

	*NCT-A Test*		*BDT Test*	

	*N*	*%*	*N*	*%*
MHE Absent	30	27.3%	18	16.4%
MHE Stage-I	21	19.1%	11	10.0%
MHE Stage-II	17	15.5%	33	30.0%
MHE Stage-III	24	21.8%	30	27.3%
MHE Stage-IV	18	16.4%	18	16.4%

## DISCUSSION

Results of this study shows significant presence of MHE in patients of compensated cirrhosis patients at 70%. Many methods are available for diagnosing MHE but all tests have some major drawbacks which include lack of standardization, requirement of expensive equipment, requirement of literacy, readily availability and cost to patient to name a few. Thus a gold standard is lacking in this area.^12^ Diagnosing MHE in illiterate is even more challenging as the most of the test require some education and constructive abilities.

Most of the previous studies have focused on reporting MHE in patients with decompensated cirrhosis, in this study we have made an effort to document MHE in patients with compensated cirrhosis. MHE in decompensated cirrhosis has been reported from 40 to 80% of patients.^5^ Variability in frequency of MHE is attributed to difference in the tests used for diagnosis. Majority of patients in our setting were illiterate and were not able to participate in more sophisticated tests for diagnosis of MHE, for this reason tests that could easily be understood and done in such population were selected.

MHE is characterized by deficits in attention, visuo-constructive abilities, visuospatial orientation, and motor function. Interpretation of psychometric tests in MHE should be done with caution in patients with comorbidities like chronic renal failure and heart failure, as they tend to overestimate MHE.^13^ About 11% of our patients were alcoholics which included 4% women showing changing trend in our population in use of liquor leading to chronic liver disease.

Major hurdles in management of MHE are awareness in clinicians regarding MHE impact on patients health and QoL, absence of gold standard tests to diagnose this condition and that treatment of this disease would result in betterment in patient condition and QoL.^14^

Although psychometric hepatic encephalopathy score (PHES) is now internationally acclaimed as favoured test^15^ but it requires that patient is able to read and write and in our country where literacy rate is low and most of patients are unable to read and write, detection of MHE using PHES is not possible. Thus we used those tests that could be carried out in these patients. Using a compromised testing protocol was a limitation of this study that might have showed skewed results but this was only possible way to detect MHE in such patients.

### Limitations of the Study

The tests for Vitamin B_12_, folate and VDRL were not included in study protocol.

### CONCLUSION

Diagnosing MHE in illiterate patients could be a challenge but with tests like NCT-A and BDT could be used in such patients.
